# Impact of an Elevated Rear Component of the Ski Binding on Joint Angles of the Lower Extremity and the Center of Mass in Recreational Skiers

**DOI:** 10.5114/jhk/194305

**Published:** 2025-06-25

**Authors:** Markus Posch, Maurice Mohr, Martin Burtscher, Klaus Greier, Julia Scharbert, Gerhard Ruedl

**Affiliations:** 1 Department of Sport Science, University of Innsbruck, Innsbruck, Austria.; 2 Department of Physical Education, University College of Education (KPH-ES), Stams, Austria.

**Keywords:** kinematic, heel height, ACL injury, recreational alpine skiing, ski binding

## Abstract

Recently, an elevated rear component of the ski binding has been associated with a reduced ACL injury risk. However, the underlying mechanism is unknown. This study aimed to evaluate the impact of elevated rear components of the ski binding on lower extremity joint angles and the center of mass (COM) in recreational skiers. To evaluate ankle, knee and hip joint angles and the COM, a cohort of 25 subjects (mean age: 25.4 ± 1.8 years) performed unipedal standing trials within a ski boot at four heel heights (position 0: +0.2 cm; position 1: +0.5 cm; position 2: +1.5 cm and position 3: +3.0 cm) on a force plate using an optical motion capture system. Repeated measures ANOVA revealed that flexion angles at the ankle joint significantly differed (η2 = 0.145) and were lowest at position 3, indicating a more neutral ankle joint (−1°). Flexion angles at the knee joint significantly increased with increasing heel height (η2 = 0.715) and were highest at position 3 (+6.9°), indicating a more flexed knee joint. Hip joint angles were not significantly different between the four different conditions (η2 = 0.082). The anterior-posterior COM position differed significantly between the four testing positions (η2 = 0.668) and was most anterior at position 3 (+3.7 cm), indicating a forward movement of the COM. An elevation of the heel component of the ski binding causes an increase in knee flexion accompanied by a forward movement of the COM, both potentially increasing hamstring co-activation as an advantageous preventive measure for ACL injuries in recreational skiing.

## Introduction

Recreational alpine skiing is a highly popular winter sports globally, yet it is linked to a significant risk of injury (Ekeland and Rodven, 2012; Kim et al., 2012). Although the risk of suffering from a skiing-related injury decreased over the past decades (Burtscher et al., 2008; Ruedl et al., 2014), the amount of skiing-related knee injuries did not decline to the same degree (Burtscher et al., 2008; Senner et al., 2013). The knee joint remains the predominant site of anatomical injury, representing approximately one-third of all injuries (Ruedl et al., 2014; Burtscher et al., 2008; Ekeland and Rodven, 2011). About 50% of recreational alpine skiers with knee injuries have sustained an anterior cruciate ligament (ACL) injury (Majewski et al., 2006), frequently accompanied by injuries to the medial collateral ligament (Posch et al., 2020).

Recreational alpine skiing is characterized by a complex interaction between the skier, the skiing equipment (ski, binding, boot, etc.), and the environment (Posch et al., 2023) (snow, slope conditions, etc.) (Hermann and Senner, 2021). Natri et al. (1999) highlighted the importance of equipment-related factors for lower extremity injury risk in recreational alpine skiing. For example, the ski length may account for more than 80% of lower extremity injuries by acting as a lever that bends or twists the knee joint (Natri et al., 1999).

Recently, Ruedl et al. (2022) investigated the influence of ski geometry data and the standing height ratio on the ACL injury risk in a large sample of recreational alpine skiers. They demonstrated that besides a reduced ski length, narrower ski widths, lower absolute standing heights at the front and rear components of the ski binding, and a lower standing height ratio (i.e., the rear component of the ski binding is more elevated compared to the front component), were linked with a reduced likelihood to suffer from an injury to the ACL (Ruedl et al. 2022). In that study, the standing height ratio was approximately 95% (an almost horizontal ski boot) in ACL-injured skiers compared to 89% (more elevated rear vs. front part of the ski binding) in uninjured skiers (Ruedl et al., 2022). Those authors speculated that an increased heel position of the ski boot could generate knee flexion (moving the knee further away from an extended position), which may be advantageous for ACL injury prevention (Ruedl et al., 2022). This speculation is based on evidence that quadriceps loading at small knee angles (close to extension) substantially elevates ACL tension, whereas the load on the ACL decreases with greater knee flexion angles (Wascher et al., 1993). Ruedl et al. (2022) further speculated that an elevated heel (at the posterior part of the ski binding) may induce a more pronounced forward lean in the skier, thereby shifting the skier’s center of gravity forward. This shift could potentially increase the applied load on the front component of the ski binding, facilitating improved ski steering (Ruedl et al., 2022). However, Ruedl et al. (2022) could only speculate why a decrease in the standing height ratio might reduce the ACL injury risk in recreational skiers and no study has examined the impact of different heel heights at the posterior part of the ski binding on joint angles and whole-body positioning so far.

Thus, this study aimed to evaluate the impact of an elevated rear component of the ski binding on joint angles of the lower extremity as well as the center of mass position in recreational skiers. We hypothesized that an elevated rear component of the ski binding would lead to more flexed knee angles and a forward shift of the center of mass.

## Methods

### Design and Procedures

Generally, the testing procedures and protocols were based on studies by Promsri et al. (2018a, 2019) but were further developed. Using a motion capture system to compute joint angles and the center of mass (COM), study participants completed a total of four unipedal standing trials within a ski boot on their dominant leg at four different heel heights:

1) position 0: the height difference between the front (toe) and the rear component (heel) was +0.2 cm;
2) position 1: the height difference between the front (toe) and the rear component (heel) was +0.5 cm;
3) position 2: the height difference between the front (toe) and the rear component (heel) was +1.5 cm;
4) position 3: the height difference between the front (toe) and the rear component (heel) was +3.0 cm.

Moreover, all trials were completed in randomized order with a rest interval of three minutes in between. Participants were given the opportunity to conduct a single practice trial to get used to the 40-s testing period. The task was to maintain a stationary stance, refraining from any unnecessary movements such as scratching or touching the stance leg with the raised leg, solely focusing on balance. Participants were instructed to gaze directly ahead at a target with a diameter of 10 cm, positioned on a wall approximately five meters away (Promsri et al., 2019). In order to adopt a ski-specific stance, participants were instructed to utilize their ski poles and to flex their hips, knees, and ankles in alignment with the alpine basic position (Wörndle et al., 2008).

Prior to commencing the measurements, written informed consent was acquired from all participants. The study was conducted in accordance with the ethical guidelines outlined in the 2008 Declaration of Helsinki. The current study protocol received approval from the Institutional Review Board (IRB) and the Board for Ethical Issues (BfEI) of the University of Innsbruck (approval code: 11/2022; approval date: 23 February 2022).

### Participants

Study participation exclusion criteria included a documented balance disorder or a medical contraindication that might influence the individual postural control. Moreover, the results of the Physical Activity Readiness Questionnaire (PAR-Q) (Warburton et al., 2011a, 2011b) were considered for exclusion criteria. The PAR-Q is a self-assessment tool designed to evaluate the safety or potential risks associated with physical exercise, taking into account factors such as health history, severity of any existing diseases and current symptoms (Warburton et al., 2011b) and therefore must have shown no abnormalities. Participants who suffered from an ACL injury in the past had to be returned to their sports for at least one year. Following the methods described by Promsri et al. (2019), leg dominance was determined by identifying the preferred leg for kicking a ball, a criterion that has been found to be most effective in previous studies for assessing interlimb differences in unipedal postural control (Promsri et al., 2018a, 2018b). Moreover, participants were asked for age, experience of skiing (total), and average skiing days per winter season. A total of 25 well-experienced recreational alpine skiers (52% females) volunteered to participate in the study, of whom 28% (n = 4 males, n = 3 females) had suffered an ACL injury in the past. The mean age of the participants was 25.4 ± 1.8 years. All participants were physically active, skied for 20.7 ± 4.7 years, and were skiing on 50.2 ± 26.9 days on average per winter season. The majority of participants (96%, n = 24) reported the right leg to be the dominant leg.

### Postural Control Tests—Instrumentations

**Figure 1:**
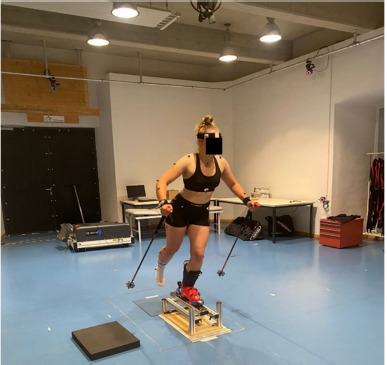
Full-Body-Plug-in Gait marker setup used in the study.

Thirty-nine reflective markers were positioned on pre-defined body segments and on the ski boots according to the “Full-Body Plug-In Gait” marker setup (Figure 1). The 3D positions of the used markers were captured by a 10-camera optical motion tracking system (Vicon Bonita 10 and Vero v2.2 cameras) using a sampling rate of 250 Hz (Vicon Motion Systems Ltd., Oxford, UK).

### Postural Control Tests—Measuring Device

**Figure 2:**
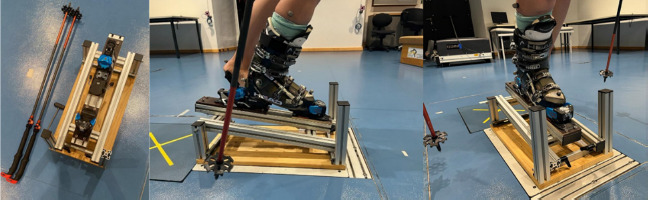
Ski binding test device fixed on a wooden plate simulating different heel positions.

A custom-made device was built to realize different heel heights during the postural control tests (Figure 2). This device was adjustable in height with adjusting screws. In addition, the ski binding was screwed firmly onto it, so that it only had to be adjusted once to the respective ski boot sole length. With the help of the adjusting screws, the correct angle was set so that the four different test positions could be simulated. The measuring device was always positioned at the same place on the force plate to be able to compare the position of the COM later. Each participant used the same type of the ski boot (Salomon S/PRO R90W) which was provided by a ski rental shop in all needed sizes.

### Postural Control Tests—Data Analysis

**Figure 3:**
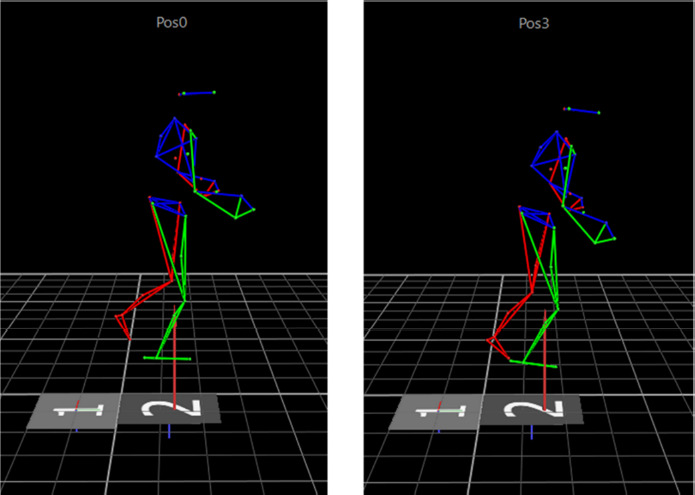
Visualization of marker trajectories in testing positions 0 and 3.

The three-dimensional marker trajectories (Figure 3) were post-processed and exported into c3d-files through Vicon Nexus software (v. 2.12, Vicon Motion Systems Ltd., Oxford, UK) and then further processed in OpenSim (v. 4.2) (Delp et al., 2007) and custom-written Matlab scripts (v. R2020a, The MathWorks, Inc., Natick, Massachusetts, US) using the OpenSim Application Programming Interface. Marker trajectories were first filtered using a third-order dual-pass low-pass Butterworth filter with a cut-off frequency of 20 Hz and then down-sampled to 125 Hz. Subsequent analyses in OpenSim included standard model scaling and inverse kinematic procedures using the generic whole-body musculoskeletal model of Catelli and colleagues (Catelli et al., 2019). The inverse kinematic analysis was completed for the middle ten seconds of each balance trial. The resulting time-dependent joint angles as well as the COM were averaged over time to compute the outcome variables for statistical comparisons between different heel heights: mean sagittal plane angles at the ankle, knee, and hip joints as well as the mean position of the COM anterior-posterior (COM-AP). Positive angles indicated ankle dorsiflexion, knee flexion, and hip flexion. Angles of zero degrees represented a neutral standing position with the ankle joint at 90° and the other joints fully extended. For better comparison between participants, the mean COM position during the position 0 trials was subtracted from the mean COM positions of the other trials resulting in a position of x = 0 (anterior (+) / posterior (-) direction) at position 0.

### Statistical Analysis

All data are expressed as means ± standard deviations. This study used a repeated measures design with four testing conditions: position 0 (+0.2 cm), position 1 (+0.5 cm), position 2 (+1.5 cm), and position 3 (+3 cm) to explore potential changes in joint angles and the anterior-posterior position of the COM between the four different testing positions. Effect sizes for ANOVA were computed as partial eta-squared (η^2^) and interpreted following guidelines outlined by Cohen (1988) as follows: (η^2^ < 0.06 indicating a small effect, 0.06 ≤ η^2^ < 0.14 indicating a moderate effect, and η^2^ ≥ 0.14 indicating a large effect). Post-hoc tests were made with matched-pairwise and Bonferroni-corrected tests. All values were normally distributed, as assessed by the Shapiro-Wilk test (*p* > 0.05). No Greenhouse Geisser correction was necessary to correct for violations of sphericity (*p* > 0.05) according to the Mauchly test for sphericity. Statistical analysis was conducted using SPSS 23.0 (IBM Corporation, Armonk, NY, USA). Two-tailed *p*-values were calculated with statistical significance set at *p* < 0.05.

## Results

Table 1 shows the comparison of joint angles between the four testing positions (0, 1, 2, 3) and heel heights among all participants.

Ankle (F(3, 72) = 4.068, *p* = 0.010, η2 = 0.145, moderate to large effect) and knee joint angles (F(3, 72) = 60.189, *p* < 0.001, η2 = 0.715, large effect) were significantly different between the four different heel heights.

Dorsiflexion angles at the ankle joint were lowest for testing position 3, indicating a more neutral ankle joint, while angles at positions 0, 1, and 2 were similar. The highest average dorsiflexion angles of the ankle were shown at testing position 1. Post-hoc analysis did not show any significant differences in dorsiflexion angles between the four testing positions (*p* > 0.05).

Flexion angles at the knee joint significantly increased with increasing heel heights and were highest at testing position 3, indicating a more flexed knee joint. The lowest average flexion angles of the knee joint were shown at testing position 0. Post-hoc analysis showed significant differences in knee flexion angles between all testing positions (*p* < 0.01).

Hip joint angles were not significantly (F(3, 72) = 3.451, *p* = 0.376, η2 = 0.126, moderate effect) different among the four different heel heights. Post-hoc analysis did not show any significant pairwise differences in hip joint angles between any testing positions (*p* > 0.05).

COM-AP differed significantly among the four testing positions (F(3, 72) = 48.323, *p* < 0.01, η2 = 0.668, large effect). The highest alteration of the COM-AP was reported at testing position 3, simulating a higher anterior forward movement of the center of mass. Post-hoc analysis showed significant differences in the COM-AP among all testing positions (*p* < 0.05).

**Figure 4:**
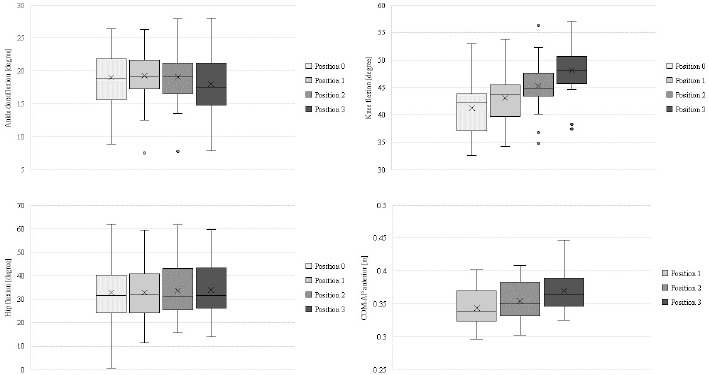
Comparison of the joint angles between the four different heel heights among all participants. *The boxplots show the median (and mean value X), interquartile range, minimum and maximum values and outliers of joint angles for the various positions*.

## Discussion

This study aimed to evaluate the potential impact of an elevated rear component of the ski binding on joint angles of the lower extremity and the center of mass position in recreational skiers. To the best of our knowledge, this is the first study considering different heel heights at the rear component of the ski binding and associated effects on joint angles and the center of mass movement during unipedal balancing. The main findings were that with increasing heel heights, knee joint flexion angles and the anterior position of the center of mass significantly increased.

### Ankle Joint

Angles at the ankle joint were highest at a slightly elevated heel position (position 1, +0.5 cm), showing a flexed position. Elevating the heel component by a maximum of 3 cm led to smaller flexion angles at the ankle joint, i.e., a more neutral position.

It has to be mentioned that in alpine skiing, flexion of the ankle joint is limited by the design and stiffness of the ski boot. In general, the design of a ski boot should always consider three essential elements: performance, comfort, and most of all safety (Knye et al., 2016).

Rigid ski boots, which restrict dorsiflexion, have the potential to transfer significant loads from the ankle and the tibia to the knee, potentially augmenting the risk of knee injuries (Hunter, 1999). Schaff and Hauser (1993) showed that stiffer ski boots could promote an upright or even a backward lean position and provoke an increased load on the knee ligaments (Hall et al., 1991). Moreover, Eberle et al. (2016) observed that stiffer ski boots, which restricted plantar flexion at the ankle joint, resulted in reduced ground reaction forces during landing situations prone to injury. This reduction further diminished the protective effect of ground reaction forces on the ACL (Eberle et al., 2016).

Generally, the flexion indices of ski boots are determined by the torque exerted around the flexion axis of the boot (Immler et al., 2019). Nominal flex indices typically range from 50 (soft) to 150 (stiff) (Immler et al., 2019). In this study, a ski boot with a flex index of 90 was used. Usually, ski boots with high flex indices are utilized by more skilled skiers whereas beginners use softer boots more often (Knye et al., 2016). Softer ski boots that might enable higher dorsiflexion angles give less resistance against external bending movements (Schaff and Hauser, 1990). Therefore, muscles in the hip, the knee, and the ankle need to generate more muscular power, leading to faster fatigue (Schaff and Hauser, 1990). In their study, Mika et al. (2012) found that prolonged use of heeled footwear could potentially lead to muscle overuse and repetitive strain injuries. Moreover, if the flexion indices are too low to limit the ankle joint movement, excessive dorsiflexion above 40° may occur likely resulting in ankle injuries (Shealy and Miller, 1989). In this study, the ankle joint angles were lower than 20°, thus no excessive dorsiflexion was shown.

An elevation of the heel component of the ski binding by +0.5 cm led to the highest average dorsiflexion angle in this study. Increasing the heel height by more than 0.5 cm led to smaller flexion angles at the ankle joint, however, post-hoc testing did not reveal significant differences for any pairwise comparison between testing positions. This might have been due to compensatory movements based on a forward shift of the COM-AP leading to more flexed knee and hip joints. Furthermore, the development of angles at the ankle joint is potentially dependent on the stiffness of the ski boot. As the average ankle angles did not differ by more than 1.3° between test positions, representing small deviations, and no excessive dorsiflexion was shown, all different heel heights possibly represent adequate positions in the underlying study when thinking about softer ski boots (Hunter, 1999; Schaff and Hauser, 1993) that should allow a certain amount of dorsiflexion (Hunter, 1999) not leading to any muscle overuse (Mika et al., 2012).

### Knee Joint

Elevating the rear component of the ski binding led to a significantly increased flexion angle at the knee joint. At testing position 3 (+3 cm), the highest flexion angles were found among all conditions. Compared to position 0, knee flexion angles were by 15% (+6.9°) higher at position 3.

Ebbeling et al. (1994) assessed the threshold at which an elevated heel height in high-heeled shoes became biomechanically detrimental. They concluded that to preserve comfort and minimize the risk of injury, women should refrain from wearing shoes with a heel height exceeding 5 cm (Ebbeling et al., 1994). These high-heeled shoes restricted the ankle range of motion and increased knee flexion (Ebbeling et al., 1994; Mika et al., 2012). These results are similar to the results of the current study, where ankle flexion angles were restricted when increasing the heel height, and knee flexion angles were highest when simulating an elevated rear component. Again, the knee joint flexion seems to be a compensatory mechanism as the COM-AP further indicated a forward shift of the center of mass.

In their case study, Rentenberger et al. (2021) recently reported of an elderly patient who received intricate lumbar spine surgery and experienced issues skiing that he addressed by mechanically shifting his center of gravity by placing a 2-cm wedge at the rear component of the ski binding. As the spine was less flexible after surgery and the lower extremities had to compensate, the patient mechanically reproduced the forward bending position with a heel lift (Rentenberger et al., 2021). In the underlying study, testing positions 1 (+ 0.5 cm) and 2 (+1.5 cm) seemed to be the most suitable heel heights that led to flexed knee joints, but did not restrict the ankle range of motion too much. A heel height increase of up to 1.5 cm would also be easy to implement in practice.

An increased heel position of the ski boot appears to elicit increased knee flexion, thereby moving the knee further away from an extended position. This observation holds considerable relevance for ACL injury prevention, as there is evidence indicating that quadriceps loading at small knee angles, near full extension, markedly increases ACL tension, while the ACL load diminishes with greater knee flexion angles (Wascher et al., 1993).

In a study by Wu et al. (2017), the level of antagonist coactivation (hamstrings) was higher among deeper knee flexion (>60°) and antagonist coactivation increased as a function of contraction intensity. They suggested that antagonist coactivation might represent a protective mechanism that served to stabilize the knee joint and maintain constant motor output (Wu et al., 2017). There is generally supportive evidence suggesting that hamstring muscles function as an agonist to the ACL and hamstring co-contraction diminishes internal rotation, anterior translation and the ACL load during weight-bearing flexion (MacWilliams et al., 1999), a finding that seems pertinent for ACL injury prevention in skiing. Therefore, an elevated heel leading to flexed knee joints and accompanying antagonist coactivation can represent an advantageous position on the skies.

### COM-AP

An elevated heel component of the ski binding significantly influenced the anterior-posterior COM position. At testing position 3 (+3 cm), the COM-AP indicated a forward movement of about 3 cm. Small heel elevations (testing positions 1 and 2) led to smaller anterior movements (0.5 and 1.5 cm). A forward displacement of the COM-AP may potentially enhance the exerted load on the front component of the ski binding, thereby facilitating improved ski steering (Ruedl et al., 2022).

In a ski-specific investigation, Koyanagi et al. (2006) examined the impact of alterations in the skiing posture on mechanical stress distribution across the knee joint. Participants, wearing ski boots, were positioned on a force plate on an artificial slope inclined at 20°, where forward and backward bending postures during single-leg stances were analyzed. An increase in muscular activity of the hamstrings and a decrease in quadriceps muscle activity was found in a forward bending position and vice versa in a backward bending posture. The hamstring muscles serve as an agonist of the ACL, and concurrent contraction of the hamstring diminishes internal rotation, anterior translation, and the ACL load during weight-bearing flexion (MacWilliams et al., 1999). Findings from a recent study by Hermann et al. (2022) demonstrated that for knee flexion angles less than 40°, simulating medium flexed knee joints, ACL loads were reduced by active hamstring activation and increased by dominant quadriceps activation in knee surrogates. Therefore, the alteration of the COM-AP in the underlying study due to the heel lift might also lead to activated hamstring muscle activity which could represent valuable information regarding injury prevention in recreational alpine skiing. Testing positions 1 (+0.5 cm) and 2 (+1.5 cm) represent possible ski-specific positions where the forward movement still occurs at a “normal” level, not leading to restrictions among ankle range of motion due to high knee flexion angles. The capability of biological organisms to produce a variety of solutions to a particular movement offers flexibility when dealing with unexpected or changing constraints (e.g., elevated heel) and therefore represents a major source of variability in movement patterns (van Wegen et al., 2002; Latash et al., 2002). During unanticipated situations on the ski slopes eccentric muscle actions are necessary to counteract passively induced loading during skiing (Hildebrandt et al., 2017). In the underlying study subjects may have used a variety of different configurations of multiple degrees of freedom (e.g., joint angles, muscle activity, etc.) to cope with the elevated heel position in their ski bindings (Promsri et al., 2021). Basically, in postural control, the goal is to maintain the body within the boundaries of the base of support (van Wegen et al., 2022), this requires the COM to remain within the boundaries of the ski boots like in the current study. By elevating the heel component of the ski binding, the COM-AP indicated a forward movement of up to 3 cm. To prevent further anterior displacement of the COM-AP, it seems that the main movement strategy appeared to produce high knee flexion angles.

### Hip

Different heel heights at the rear component of the ski binding did not have any significant influence on the hip joint angles. The highest flexion angles of the hip joint were found at position 3. Generally, the hip joint was flexed in all different testing positions which seemed to be in line with the specifications of the Alpine basic position (Wörndle et al., 2008). The aim of the Alpine basic position, which is part of the “Austrian Ski Instruction Teaching Curriculum”, aims to assume a body position that is ready to move so that you can react quickly in all directions in every phase of alpine skiing (Wörndle et al., 2008). The ankle, knee, and hip joints and the spine are in a medium, flexed position that is ready for movement (Wörndle et al., 2008). The subjects in the present study were experienced skiers with about 20 years of skiing experience on average. Therefore, it can be assumed that the subjects tried to apply the Alpine basic position during the testing procedures.

Recently, Ruedl et al. (2022) showed that a standing height ratio (ratio between the front and rear component of the ski binding) was found to be an independent equipment-related risk factor for an ACL injury in recreational skiing. Within the group of uninjured controls, the mean absolute differences between the standing height at the front and rear components of the ski binding were 5 mm (Ruedl et al., 2022). This difference within the standing height of 5 mm is equivalent to testing position 1, where the heel was elevated by 0.5 cm. As already mentioned, testing positions 1 and 2 seem to be advantageous skiing-specific positions where the ankle, knee, and hip joints are in a medium flexed position, not restricting the ankle range of motion leading to a more forwarded position. This seems to be a requirement for getting into an Alpine basic position. According to Wörndle et al. (2008), alpine skiing behavior describes a constant reaction to external forces in every situation based on flexed joints and a forward bending posture. Solid alpine skiing behavior further enables safe gliding on slopes and creates the conditions for edging and steering appropriate to the situation (Wörndle et al., 2008).

### Limitations

One limitation of the study is the applicability of the computed postural tests from controlled laboratory settings to on-slope field conditions. The study was conducted in a controlled laboratory environment with standardized conditions, and thus, the results of the postural tests may vary in field settings. The flex indices of the ski boot influence the development of dorsiflexion. In the underlying study, a standardized ski boot with a flex index of 90 (medium) was used among all participants to make results comparable. Moreover, the skill level of the skier could represent a potential limitation as the subjects in the present study were experienced skiers. Experienced and more skilled skiers might try to apply the Alpine basic position assuming a body position that is ready to move quickly during the testing procedures, while less skilled skiers might use different strategies to compensate the different heel heights. Less skilled skiers might not use knee joint flexion as a compensatory mechanism or generate movements that might lead to a backward shift of the COM-AP. However, to the best of our knowledge, this is the first study that considered different heel heights at the rear component of the ski binding leading to potentially different joint angles and forward movement regarding the whole-body positioning.

## Conclusions

In conclusion, an elevation of the heel component of the ski binding causes an increase in knee flexion accompanied by a forward movement of the center of mass, both potentially increasing hamstring co-activation as an advantageous preventive measure for ACL injuries in recreational skiing.
